# PPAR Activation Induces M1 Macrophage Polarization via cPLA_**2**_-COX-2 Inhibition, Activating ROS Production against *Leishmania mexicana*

**DOI:** 10.1155/2013/215283

**Published:** 2013-02-28

**Authors:** J. A. Díaz-Gandarilla, C. Osorio-Trujillo, V. I. Hernández-Ramírez, P. Talamás-Rohana

**Affiliations:** Departamento de Infectómica y Patogénesis Molecular, Centro de Investigación y de Estudios Avanzados del IPN, Avenida Instituto Politécnico Nacional No. 2508, Colonia San Pedro Zacatenco, Delegación Gustavo A. Madero, 07360 México, DF, Mexico

## Abstract

Defence against *Leishmania* depends upon Th1 inflammatory response and, a major problem in susceptible models, is the turnoff of the leishmanicidal activity of macrophages with IL-10, IL-4, and COX-2 upregulation, as well as immunosuppressive PGE_2_, all together inhibiting the respiratory burst. Peroxisome proliferator-activated receptors (PPAR) activation is responsible for macrophages polarization on *Leishmania* susceptible models where microbicide functions are deactivated. In this paper, we demonstrated that, at least for *L. mexicana*, PPAR activation, mainly PPAR**γ**, induced macrophage activation through their polarization towards M1 profile with the increase of microbicide activity against intracellular pathogen *L. mexicana*. PPAR activation induced IL-10 downregulation, whereas the production of proinflammatory cytokines such as TNF-**α**, IL-1**β**, and IL-6 remained high. Moreover, PPAR agonists treatment induced the deactivation of cPLA_2_-COX-2-prostaglandins pathway together with an increase in TLR4 expression, all of whose criteria meet the M1 macrophage profile. Finally, parasite burden, in treated macrophages, was lower than that in infected nontreated macrophages, most probably associated with the increase of respiratory burst in these treated cells. Based on the above data, we conclude that PPAR agonists used in this work induces M1 macrophages polarization via inhibition of cPLA_2_ and the increase of aggressive microbicidal activity via reactive oxygen species (ROS) production.

## 1. Introduction

Leishmaniasis is a collection of parasitic diseases caused by two dozens species of protozoa belonging to the genus *Leishmania* and spread by the bite of a sandfly. Two main clinical forms are known: cutaneous leishmaniasis, affecting the skin causing scars and eventually disfiguration, and systemic or visceral leishmaniasis that can lead to fatal complications if untreated [1].

In México, *Leishmania mexicana* is the causative agent of two forms of cutaneous leishmaniasis. Localized cutaneous leishmaniasis (LCL) is characterized by ulcerative skin lesions that develop at the site of the bite of the sandfly; diffuse cutaneous leishmaniasis (DCL), which consists of nonulcerative nodules that spread throughout the skin, leads to severe mutilation because of the invasion of naso- and oropharyngeal mucosa [2, 3]. Peroxisome proliferator-activated receptors (PPARs) are ligand-activated transcription factors expressed in macrophages, where they control the inflammatory response; there are three isoforms, PPAR*α*, PPAR*β*/*δ*, and PPAR*γ*, that exhibit different tissue distribution as well as different ligand specificities [4]. PPAR*γ* promotes the differentiation of monocytes into anti-inflammatory M2 macrophages in humans and mice while the role of PPAR*β*/*δ* in this process has been reported only in mice, and no data are available for PPAR*α* [5].

Differential cytokine production is a key feature of polarized macrophages; while Th1 cytokines promote proinflammatory M1 macrophages, Th2 cytokines support an “alternative” anti-inflammatory M2 macrophage phenotype. Modulation of proinflammatory cytokines by *Leishmania* species *in vitro* and *in vivo* is reported elsewhere [6]. In general, *Leishmania* infections induce tumor necrosis factor (TNF-*α*) production; interleukin-1*β* (IL-1*β*) generation is abrogated by *L. donovani* infection *in vitro* and *in vivo*, whereas it is induced by *L. major* infection. These observations indicate that different species of *Leishmania* can differentially modulate the proinflammatory cytokines. In addition, it is now well documented that these cytokines play a decisive role in the modulation of chemokines, which are recognized for their function in cell recruitment and promotion of the inflammatory reaction [6].

Regarding anti-inflammatory cytokines, recent studies have demonstrated the critical role of IL-10 in susceptibility to cutaneous and visceral leishmaniasis caused by different *Leishmania* species such as *L. major, L. donovani*, *L. mexicana*, and *L. amazonensis*. IL-10 suppresses IFN-*γ* synthesis by inhibiting accessory cell functions and also can reduce the production of Nitric Oxide (NO) by activated macrophages. IL-10 also downregulates the expression of MHC class I and class II molecules as well as costimulatory B7 molecules on macrophages. Moreover, a recent study has shown that IL-10-deficient BALB/c mice can control infection with *L. major* suggesting that IL-10 plays a key role in mediating the susceptibility and pathogenesis of cutaneous leishmaniasis [7, 8].

Prostaglandins are often associated with anti-inflammatory activities such as inhibition of effector functions of inflammatory cells. These include inhibition of mediator release from macrophages, neutrophils, mast cells, basophils, and lymphocytes; they can also downregulate macrophage functions, particularly, prostaglandin E_2_ (PGE_2_) [9], which is synthesized throughout the duration of the inflammatory response, largely via the sequential activities of cytosolic phospholipase A_2_ (cPLA_2_), cyclooxygenase-2 (COX-2; rate-limiting enzyme), and microsomal PGE synthase-1 (mPGES-1). This pleiotropic prostanoid serves as an underlying modulator of inflammation by mediating and modulating cytokine-target gene expression at transcriptional and posttranscriptional/translational levels [10–13].

The cPLA_2_ and COX-2 promoters contain a PPAR response element (PPRE); thus, PPAR*γ* agonists including anti-inflammatory drugs may affect COX-2 and cPLA_2_ transcription and expression. Pérez-Santos and Talamás-Rohana [14] demonstrated that indomethacin (INDO) administration induced the intracellular killing of *L. mexicana* parasites in infected BALB/c mice; these results suggest that suppression of PGs by INDO promotes the development of a protective Th1 type response in susceptible mice by enhancement of IL-12, IFN-*γ*, and NO production.

Classically activated macrophages have a high capacity to present antigens and to produce IL-6, IL-1*β*, TNF-*α*, and toxic intermediates (NO and ROS), consequently, orienting the immune system to a polarized type I response. The various life-cycle stages of *Leishmania* have different sensitivities to ROS and elicit different oxidative responses of the macrophage. *Leishmania* protects itself against the macrophage's oxidative burst through the expression of antioxidant enzymes and proteins, as well as actively by the inhibition of NO and ROS production in the macrophage [15].

In the present work, we analyzed the effect of PPAR's agonists during the early-time infection of J774A.1 macrophages with *L. mexicana* and addressed the issue of whether the addition of PPAR agonists to J774A.1 macrophages infected with *L. mexicana* could increase ROS production by polarization of M2 towards M1 macrophages, inhibiting cPLA_2_ and COX-2 enzymes.

## 2. Material and Methods

### 2.1. Antibodies and Reagents

PPAR*β*/*δ* and PPAR*γ* antibodies were purchased from Cayman Chemical (Ann Arbor, MI, USA); cPLA_2_, p-cPLA_2_ (Ser^505^), COX-2, MR/CD206, and p44/42 MAP kinase (ERK1/2) antibodies were from Santa Cruz Biotechnology Inc. (Santa Cruz, CA, USA); TLR4/CD284 antibody was from IMGENEX (San Diego, CA, USA). Anti-mouse IgG-conjugated horseradish peroxidase was from Pierce Biotechnology, Inc., (Rockford, IL, USA), and anti-rabbit IgG-conjugated horseradish peroxidase was from Zymed Laboratories (San Francisco, CA, USA). PPAR*β*/*δ* agonist (GW501516) and PPAR*γ* agonist (GW1929) were obtained from Alexis Biochemicals (ENZO Life Sciences, Inc. Ann Arbor, MI, USA); cPLA_2_ inhibitor (arachidonyl trifluromethyl ketone (ATK)) was obtained from Cayman Chemical. All D-MEM and RPMI-1640 media were purchased from Gibco-BRL, Life Technologies (Grand Island, NY, USA). Fetal bovine serum (FBS) was from PAA Laboratories (GE, Healthcare, UK). All materials for SDS-PAGE were purchased from Bio-Rad. Lipopolysaccharide (LPS, *Escherichia coli* serotype 0111:B4) and all the other chemicals and biochemicals were from Sigma-Aldrich (St. Louis, MO, USA). Prostaglandin E_1_ (PGE_1_), prostaglandin F_1*α*_ (PGF_1*α*_), 6-ketoprostaglandin F_1*α*_ (6-keto-PGF_1*α*_), prostaglandin E_2_ (PGE_2_), prostaglandin F_2*α*_ (PGF_2*α*_), and deuterated prostaglandins (^2^H) were purchased from Cayman Chemical. HPLC grade solvents, glacial acetic acid, acetonitrile, methanol, chloroform and, all the other chemicals were from Sigma-Aldrich. Solid-phase extraction (SPE) cartridges (C18) were purchased from Millipore (Milford, MA, USA).

### 2.2. Parasites

*Leishmania mexicana* (MHOM/MX/92/UAY68) promastigotes were grown at 26°C in RPMI-1640 medium supplemented with 10% heat-inactivated FBS, 2 mM L-glutamine, 100 IU/mL penicillin, 100 *μ*g/mL streptomycin, and 10 mM HEPES. Promastigotes were used at the stationary phase of growth.

### 2.3. Cell Culture

Murine macrophage cell line J774A.1 (American Type Culture Collection, Rockville, MD, USA) was cultured at 37°C in humidified 5% CO_2_/95% air in DMEM containing 10% heat-inactivated FBS, 2 mM L-glutamine, 100 IU/mL penicillin, and 100 *μ*g/mL streptomycin. Cells were incubated for 24 h before being used for the required assays. For all experiments, cells were grown up to 80–90% confluence, and then the medium was replaced with a fresh medium, and cells were incubated with *L. mexicana*. Cells were not subjected to more than 20 cell passages.

### 2.4. Infection of Macrophages

J774A.1 macrophages (1 × 10^6^/well) were cultured in 24-well culture plates. Cells were incubated with promastigotes of* L. mexicana* at a ratio of 20 parasites per macrophage or treated with LPS (1 *μ*g/mL) for the indicated periods, after which noningested promastigotes were washed off with warm D-MEM. Where indicated, cells were also pretreated for 24 h with pharmacological agonists GW501516 (100 nM), GW1929 (600 nM) and for 1 h with ATK (75 *μ*M), prepared in DMSO or ethanol. Vehicle controls were included in each experiment.

### 2.5. Real-Time PCR Assays

The total mRNA from noninfected, *L. mexicana*-infected, or LPS-stimulated J774A.1 macrophages was extracted with TRIzol reagent (Life Technologies Corporation, USA) according to the manufacturer's instructions. The retrotranscription reaction was performed with the First Strand cDNA Synthesis Kit according to the manufacturer's instructions (Fermentas Life Sciences, USA) in an iCycler Thermal Cycler (Bio-Rad). RT-PCR amplifications were performed as described by Estrada-Figueroa et al. [16]. Reactions were done in a real-time PCR 7500 apparatus (Applied Biosystems) in a final volume of 20 *μ*L using 100 ng of cDNA and 10 *μ*L of TaqMan Universal Master Mix II (Applied Biosystems, Foster City, CA, USA). Primers were from Applied Biosystems (TaqMan Gene Expression Assay): TNF-*α* (Mm00443258_m1), IL-1*β* (Mm01336189_m1), IL-6 (Mm00446190_m1), COX-2 (PTGS2; Mm00478374_m1), IL-10 (Mm00439614_m1), and *β*-Actin (Mm00607939_s1), with the following conditions: 50°C for 2 min, then 95°C for 10 min, followed by 40 cycles at 95°C for 15 sec and 60°C for 1 min. To verify results, each sample was analyzed in quadruplicate. Levels of transcription were normalized to those of *β*-actin (internal standard) to determine the variability in the amount of cDNA in each sample. With the C_T_ values obtained, the 2^−ΔΔC_T_^ method was followed to calculate the level of expression of each cytokine or mediator gene in treated macrophages in comparison with the expression level of the same cytokines or mediators in the nontreated macrophages, according to the formula [17]:

(1) ΔΔCT=(CT  target−CTβ  actin)  treated−(CT  target−CTβ  actin)  nontreated.

### 2.6. Preparation of Cell Extracts and Western Blot Analysis

Macrophages were pretreated for 24 h with the PPAR*β*/*δ* and PPAR*γ* agonists or for 1 h with cPLA_2_ inhibitor ATK, while some (basal control) were not. After incubation conditions (noninfected, *L. mexicana*-infected, or 2 h LPS-stimulated macrophages, where indicated), cells were quickly washed twice with icecold PBS and lysed by scraping in RIPA buffer (50 mM Tris-HCl, pH 7.4, 150 mM NaCl, 1% Nonidet P-40, 1 mM PMSF, 1 *μ*g/mL aprotinin, 1 *μ*g/mL leupeptin, 1 mM EDTA, 1 mM NaF, and 1 mM Na_3_VO_4_). Lysates were centrifuged at 10,000 ×g for 15 min at 4°C to yield the whole cell extract. Supernatants were transferred to fresh tubes and stored at −70°C until required. Protein concentration was determined using a BCA protein assay with bovine serum albumin as standard. Equal amounts of total cell lysates (60 *μ*g protein) were solubilized in sample buffer by boiling for 5 min, separated on 10% SDS-PAGE, and then transferred onto a nitrocellulose membrane using a Trans blot system (Bio-Rad). Nitrocellulose membranes were then incubated successively in TBST blocking buffer (50 mM Tris-HCl pH 7.4, 150 mM NaCl (TBS)) containing 5% skimmed dried milk and 0.05% Tween 20 for 1 h at room temperature, to block nonspecific protein binding. Membranes were incubated overnight at 4°C with a specific anti-PPAR*β*/*δ* (1 : 500), anti-PPAR*γ* (1 : 500), anti-phospho-cPLA_2_ (1 : 500), total cPLA_2_ (1 : 500), or anti-COX-2 (1 : 500), antibodies in TBST. Membranes were washed with TBST five times for 5 min each and incubated with the appropriate Horseradish peroxidase-conjugated secondary antibody (1 : 1000) for 1 h at room temperature. Blots initially probed with an antibody were stripped by incubation in 50 mM Tris-HCl pH 6.7, 100 mM *β*-mercaptoethanol, and 2% SDS for 30 min at 50°C. Following extensive washing, blots were reprobed with an anti-ERK1/2 antibody (1 : 5000) as a loading control. Immunoreactive proteins were visualized by enhanced chemiluminescence detecting system. Densitometry analyses of immunoblots were performed using Syngene GeneGenius scanning densitometer and software.

### 2.7. Phagocytic Assays

A flow cytometry-based method was used to study the phagocytic activity of macrophages. J774A.1 macrophages were seeded at 1 × 10^6^ cells/mL per well in 24-well tissue culture plates and incubated at 37°C, 5% CO_2_ for 24 h. Macrophages were treated or not with PPAR agonists (24 h) or cPLA_2_ antagonist (1 h) and incubated with (FITC)-conjugated zymosan A BioParticles (Molecular Probes Europe BV, Leiden, The Netherlands) at 50 particles/cell ratio for 2 h, unless indicated otherwise, or 10 *μ*M CFSE-labeled promastigotes (1 : 10 ratio) for 60 or 120 min at 37°C except control wells. After incubation, excess nonphagocytized promastigotes or particles were removed by washing. Cells were collected in tubes, and phagocytosis was determined by two criteria: (1) the number of phagocytizing cells and (2) the mean fluorescent intensity (MFI) in a FACSCalibur.

### 2.8. Expression of Mannose Receptor and Toll-Like Receptor 4 (TLR4)

Macrophages were pretreated or not for 24 h with the PPAR*β*/*δ* and PPAR*γ* agonists or for 1 h with cPLA_2_ inhibitor ATK. After incubation conditions: noninfected, *L. mexicana*-infected, LPS or zymosan-stimulated macrophages, where it is indicated, cells were quickly washed twice with icecold PBS containing 2% of FBS (FACS buffer) and scraped on FACS buffer; then, cells were spin down, and the supernatant was removed. After that, cells were resuspended in 1 mL of fixer solution and incubated for 1 h at 37°C; then, cells were centrifuged and washed twice with FACS buffer. Nonspecific staining was blocked with 10% PBS-goat serum for 1 h at 37°C (Fc block). After two washes with FACS buffer, the appropriate antibodies were added, rabbit anti-human MR (1 : 100), and mouse anti-human TLR4 (1 : 100) and incubated for 1 h at 37°C in FACS buffer. After washing the samples twice, they were incubated with the appropriate antibody: goat anti-rabbit IgG (H + L), rhodamine-conjugated antibody (1 : 100; Millipore), donkey anti-mouse IgG (H + L), and Pacific blue-conjugated antibody (1 : 100; Sigma-Aldrich) for 1 h at 37°C. Finally, samples were washed twice and read in a FACSCalibur.

### 2.9. Oxidative Metabolism

The oxidative metabolism of J774A.1 macrophages was measured by their ability to reduce yellow-colored nitroblue tetrazolium (NBT) to blue formazan, through the production of superoxide anions as described by Nessa et al. [18]. Macrophages were simultaneously incubated with promastigotes and NBT (1 mg/mL in PBS) for the indicated times. To determine if PPAR agonists were able to increase oxidative metabolism, macrophages (2 mL, 1 × 10^6^/mL) were allowed to adhere to coverslips in plastic Petri dishes (35 × 10 mm; Nunclon, Denmark) by incubation at 37°C for 24 h with PPAR agonists or cPLA_2_ antagonist for 1 h. Then, macrophages were infected with *L. mexicana* promastigotes (1 : 10 ratio). After that, 1 mL of NBT solution was added to the reaction mixture and incubated at 37°C for the indicated times. The reaction was stopped by adding 1 mL of 0.5% HCl, and cells were further stained with fuccina for 30 sec. Then, they were washed three times with PBS, and positive-oxidative burst cells were counted in a light microscope (100 cell/field and six fields/condition). Quantitative production of formazan was determined in 96-well plates [19]; macrophages (1 × 10^5^) were stimulated with PPAR agonists as described above, and then they were infected with promastigotes for the indicated periods and NBT added. At the end of each time, macrophages were washed with 70% methanol in order to remove nonreduced NBT, the produced formazan was dissolved in DMSO and the optical density of the solution was measured in a spectrophotometer (Bio-Rad) at 630 nm wavelength. A Petri dish or a 96-well plate with noninfected macrophages was incubated with NBT and served as control for each type of experiment.

### 2.10. Prostaglandins Extraction

The following procedure was developed for the separation of eicosanoids from 24-well cell culture plates containing 2 mL of media. Media was collected and centrifuged for 5 min at 10,000 xg to remove cellular debris. Produced eicosanoids were isolated via solid-phase extraction using SPE cartridge C_18_ from Millipore. Columns were prewashed with 2 mL of MeOH followed by 2 mL of H_2_O. After applying the sample to the columns, they were washed with 1 mL of 10% MeOH, and prostaglandins were eluted with 1 mL of MeOH. The eluate was dried under vacuum and redissolved in 100 *μ*L of chloroform-MeOH (2 : 1).

### 2.11. Mass Spectrometry (MS)

All MS analyses were performed using an Applied Biosystems 3200 QTRAP hybrid, triple-quadrupole, linear ion trap mass spectrometer equipped with a Turbo V ion source and operated in MRM mode. For all experiments, the Turbo V ion source was operated in a negative electrospray mode with N_2_ gas, and the QTRAP parameters DP, EP, CE, and CXP were set and maximized for each eicosanoid, and all the samples were loaded by direct infusion at 10 *μ*L/min.

### 2.12. Statistical Analysis

To take into account all values of the kinetics of macrophages infection, a statistical analysis was performed using two-way ANOVA and Bonferroni's multiple comparison tests, using GraphPad Prism 4.0 (GraphPad Software, San Diego, CA). Differences with *P* < 0.05 were considered significant.

## 3. Results

### 3.1. PPAR Agonists Downregulate cPLA_**2**_ and COX-2 Expression in J774A.1 Macrophages Infected with *Leishmania mexicana* Promastigotes

**Figure 1:**
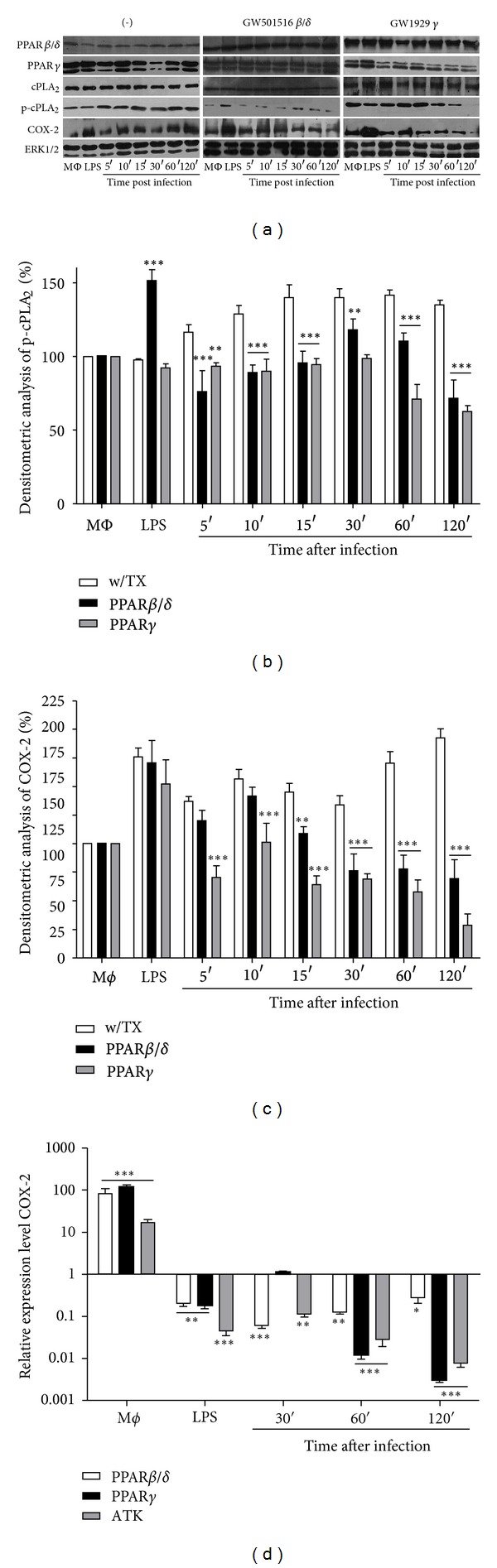
PPAR agonists inhibit cPLA_2_ phosphorylation and COX-2 expression in *L. mexicana*-infected macrophages. (a) Protein expression for PPARs and COX-2 and cPLA_2_ phosphorylation levels were evaluated by Western blotting. (b) Densitometry analyses of cPLA_2_ phosphorylation and (c) COX-2 expression were performed in basal conditions as well as in macrophages treated or not with PPAR agonists. (d) COX-2 mRNA expression was evaluated by qRT-PCR and analyzed by 2^−ΔΔC_T_^ method. Total ERK1/2 was probed to normalize protein loading. Results are representative of three independent experiments. Graph bars are mean ± SEM of three independent experiments, and statistical analysis was done comparing, for each time, treated versus nontreated macrophages; (*) *P* < 0.05, (**) *P* < 0.01, and (***) *P* < 0.001.

In *L. mexicana*-infected macrophages, the activation of cPLA_2_ by phosphorylation and the expression of COX-2 are triggered. The activation of these enzymes is considered necessary within the proinflammatory response, whereas PPAR activation is considered as an important part of the anti-inflammatory process, both *in vivo* and *in vitro* [20–22]. To examine the possibility that PPAR agonists could inhibit cPLA_2_ phosphorylation and COX-2 expression, J774A.1 macrophages were incubated with different PPAR agonists, and then cells were infected with *L. mexicana* promastigotes. The effects of *L. mexicana* promastigotes on the expression of PPAR, COX-2, and cPLA_2_ phosphorylation in J774A.1 macrophages were examined (Figure 1). Results showed that there were no changes in the expression of PPARs after infection with *L. mexicana* promastigotes of macrophages, whether treated or not with PPAR agonists; however, cPLA_2_ phosphorylation diminished significantly through the infection when macrophages were treated with PPAR agonists (Figures 1(a) and 1(b)); in addition, COX-2 protein also diminished significantly after infection (Figures 1(a) and 1(c)); COX-2 mRNA expression was strongly upregulated after *L. mexicana* infection (see Figure 1 available in Supplementary Material online at http://dx.doi.org/10.1155/2013/215283), but it was downregulated in a time-dependent manner (Figure 1(d)), when J774A.1 macrophages were treated with PPAR agonists and infected with *L. mexicana* promastigotes.

### 3.2. PPAR Agonists Downregulate IL-10 and Sustain Proinflammatory Cytokines Expression after Infection

**Figure 2:**
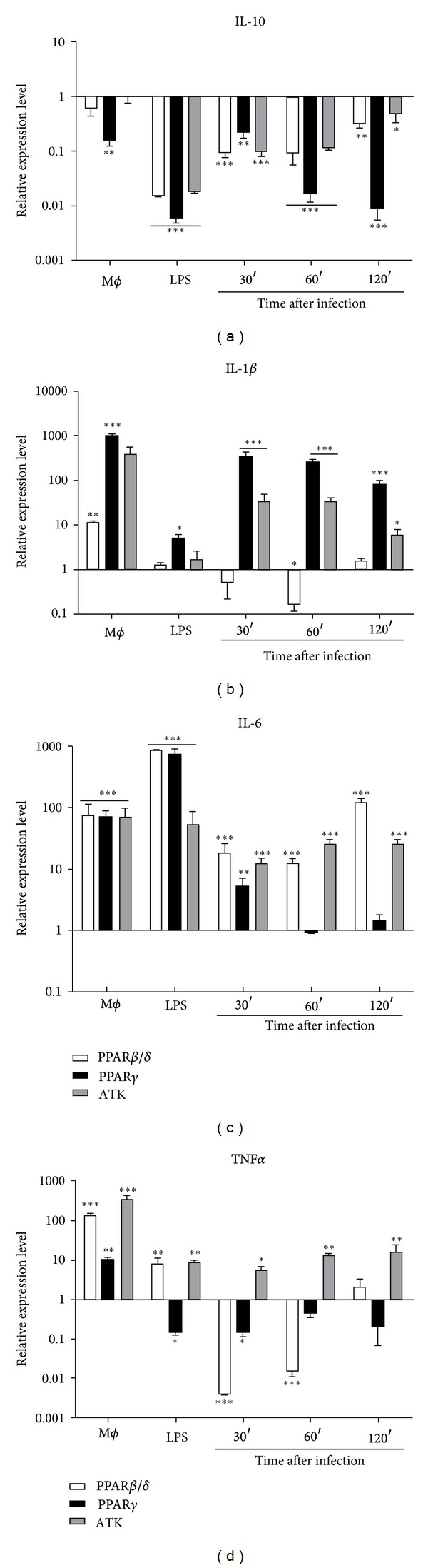
Cytokine determination in *L. mexicana*-infected macrophages. Levels of gene expression for each sample were normalized with *β*-actin RNA as internal control. Modulation was expressed relative to the untreated control using the 2^−ΔΔC_T_^ method. The *x*-axis intercepts the *y*-axis at “1” to show the increase and the decrease of each cytokine compared to nontreated infected macrophages. Relative expression level for each cytokine was calculated according to ΔΔC_T_ = (C_T_ test − C_T_ *β*-actin) treated − (C_T_ test − C_T_ *β*-actin) untreated formula [17]. Graph bars are mean ± SEM of three independent experiments, and statistical analysis was done comparing, for each time, treated versus nontreated macrophages; (*) *P* < 0.05, (**) *P* < 0.01, and (***) *P* < 0.001.

Cytokines and microbial products profoundly and differentially affect the function of mononuclear phagocytes. It is well established that different species of *Leishmania* can differentially modulate important inflammatory response mediators [23]. In order to see how PPAR agonists could modulate the inflammatory response on J774A.1 macrophages infected with *L. mexicana*, we evaluated the transcripts of some inflammatory cytokines and anti-inflammatory IL-10. The capacity of these macrophages to produce TNF-*α*, IL-1*β*, IL-6, and IL-10 cytokines in response to *L. mexicana* promastigotes was tested via qRT-PCR (Figure 2). The infection of J774A.1 macrophages resulted in an increase in TNF-*α*, IL-1*β*, and IL-6 expression; IL-10 was downregulated during *Leishmania* infection, although its expression recovered compared to noninfected macrophages (Supplementary Figure 2). However, when noninfected macrophages were previously treated with PPAR*β*/*δ* agonist (white bars), proinflammatory IL-6 was upregulated, and its expression was held during infection; TNF-*α* and IL-1*β* were overexpressed, but they were downregulated at 30 to 60 min (after infection), and its expression recovered at 120 min (after infection). Moreover, when noninfected macrophages were treated with PPAR*γ* agonist (black bars), TNF-*α* was upregulated, but its expression was downregulated after infection; overregulation of IL-6 diminished after infection, but it was held equivalent to nontreated macrophages; IL-1*β* was upregulated, and its expression was held during infection. Anti-inflammatory IL-10 cytokine was downregulated with both PPAR agonists after infection. On the other hand, when cPLA_2_ was blocked 1 h before the infection by treatment of macrophages with cPLA_2_ antagonist ATK (gray bars), proinflammatory cytokines TNF-*α*, IL-1*β*, and IL-6 were upregulated; furthermore, IL-10 expression was significantly affected when cPLA_2_ was inhibited. In summary, PPAR agonists and cPLA_2_ antagonist set down the levels of IL-10; at the same time they upregulated TNF-*α*, IL-1*β*, and IL-6 cytokines, or at least they were held overexpressed after infection as occurred with nontreated infected macrophages (Supplementary Figure 2), all together are evidence of a possible M2 to M1 polarization.

### 3.3. PPAR Activation and cPLA_**2**_ Inhibition Induce TLR4 Expression in *L. mexicana*-Infected Macrophages

**Figure 3:**
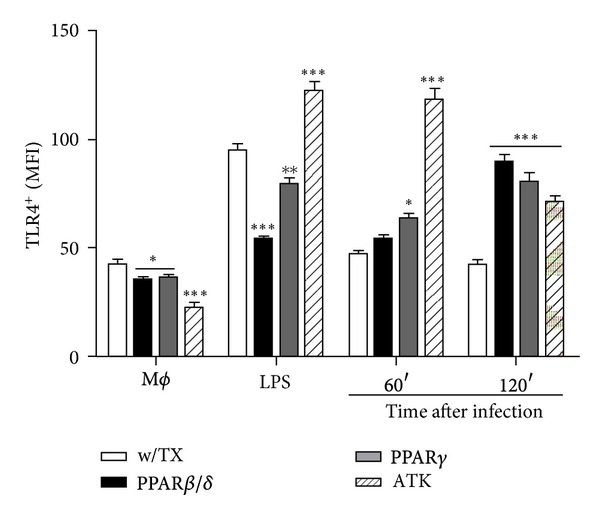
TLR4 expression in *L. mexicana*-infected macrophages. Cells were treated or not with PPAR agonists for 24 h and cPLA_2_ antagonist for 1 h before infection. TLR4 expression was analyzed by flow cytometry. LPS (2 h) was used as a positive control of induction. Graph bars are mean ± SEM of three independent experiments, and statistical analysis was performed comparing, for each time, treated versus non-treated macrophages; (*) *P* < 0.05, (**) *P* < 0.01, and (***) *P* < 0.001.

Polarized macrophages differ in terms of receptor expression, cytokine and chemokine repertoires, and effector function. M1 macrophages exposed to the classic activation signals express receptors such as CD16, CD32, CD64, TLR2, and TLR4, whereas M2 macrophages are characterized by abundant levels of nonopsonic receptors such as the mannose receptor (MR) [24]. In order to investigate if PPAR agonists and cPLA_2_ antagonist induced macrophage polarization during *L. mexicana* infection, we evaluated MR and TLR4 expression by flow cytometry. Since BALB/c mice macrophages can fully support maturation of alternatively activated macrophages, J774A.1 macrophages were incubated with PPAR agonists and cPLA_2_ antagonist, and then infected or stimulated with LPS and zymosan; neither infection nor treatments induced MR expression, an M2 receptor classified as an alternative activation marker (Supplementary Figure 3); however, PPAR agonists and cPLA_2_ antagonist induced an increase of TLR4 expression at 60 and 120 min after infection (Figure 3), an M1 receptor classified as classical activation marker. These results show that PPAR*β*/*δ* GW501516 and PPAR*γ* GW1929 agonists and cPLA_2_ antagonist do not help to keep the M2 polarization profile; instead, PPAR agonists and cPLA_2_ antagonist promote the polarization of macrophages toward an M1 profile.

### 3.4. PPAR*γ* Activation by Agonist and cPLA_**2**_ Inhibition Reduce Parasite Burden

**Figure 4:**
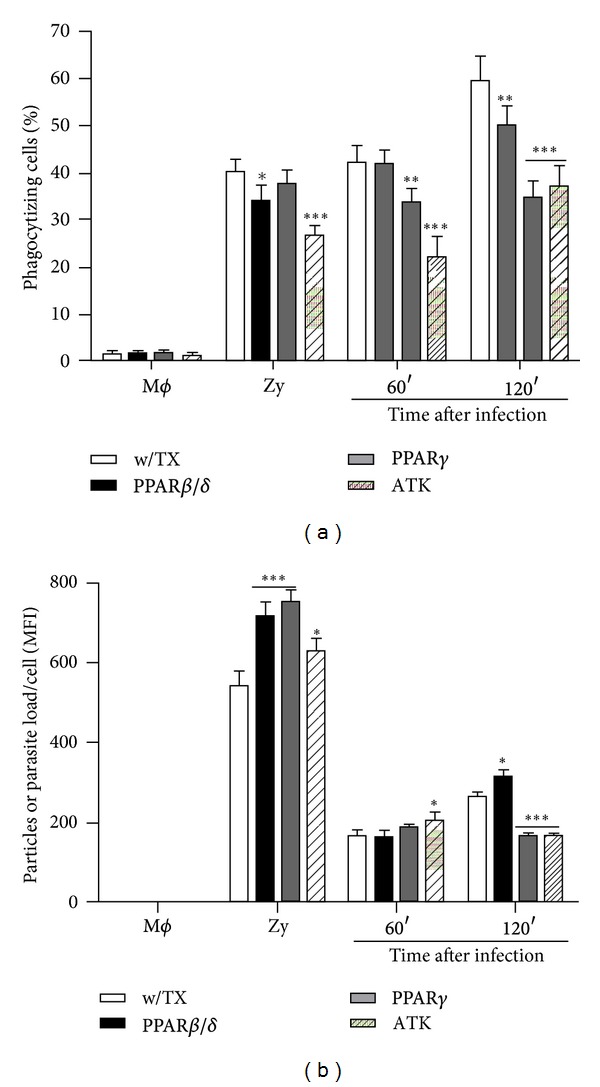
Phagocytic activity of J774A.1 macrophages was determined for zymosan and *L. mexicana* promastigotes uptake; treated or nontreated macrophages were incubated with zymosan-FITC for 2 h or infected with CFSE-promastigotes for 1-2 h. (a) Phagocytizing macrophage percentage. (b) Zymosan particles or parasites/cell (MFI). Graph bars are mean ± SEM of three independent experiments and statistical analysis was done comparing, for each time, treated versus nontreated macrophages; (*) *P* < 0.05, (**) *P* < 0.01, (***) *P* < 0.001.

PPAR**γ** expression is strongly associated with maturation of M2 macrophages. Gallardo-Soler et al. and Adapala and Chan [25, 26] have demonstrated that PPAR agonists increased intracellular growth of *L. major* in bone-marrow-derived macrophages; moreover, PPAR*γ* agonist, Curcumine, induced PPAR**γ** expression in residential, liver, and spleen macrophages of BALB/c mice. In addition, oral administration of Curcumin further increases PPAR**α** and PPAR**γ** expression, and this increase was associated with a heavier parasite burden. To analyze how PPAR*β*/*δ* GW501516 and PPAR*γ* GW1929 agonists and cPLA_2_ inhibition affected parasite burden in treated macrophages, their phagocytic activity was evaluated through two parameters: the phagocytizing cell percentage and the number of phagocytized zymosan particles or parasites/cell; treated or nontreated macrophages were incubated with FITC-labeled zymosan particles by 2 h or infected with CFSE-labeled promastigotes by 1-2 h (Figure 4). PPAR*γ* GW1929 agonist does not increase the number of zymosan-phagocytizing macrophages, but PPAR*β*/*δ* GW501516 agonist and cPLA_2_ ATK antagonist treatments decreased the number of zymosan-phagocytizing macrophages (*P* < 0.05 and *P* < 0.001, resp.; Figure 4(a) (zymosan)); on the contrary, zymosan particles/cell (mean fluorescence intensity (MFI)) increased significantly with all treatments, GW501516 and GW1929 (*P* < 0.001) and ATK (*P* < 0.05) (Figure 4(b) (zymosan)). This result shows that phagocytic activity *per se* was not affected by treatments. The number of CFSE-labeled parasites-phagocytizing macrophages did not increase by treatments either; instead, parasites-phagocytizing macrophages diminished significantly with GW1929 (*P* < 0.01) and ATK (*P* < 0.001) at 60 min after infection and 2h after infection, GW501516 (*P* < 0.01), GW1929 (*P* < 0.001), and ATK (*P* < 0.001) (Figure 4(a)). Only cPLA_2_ antagonist (Figure 4(b)) slightly increased the number of parasites/cell (*P* < 0.05) at 60 min after infection, but neither PPAR*β*/*δ* nor PPAR*γ* agonists increased it; however, at 120 min after infection PPAR*β*/*δ* agonist slightly increased parasite burden (*P* < 0.05), but PPAR*γ* agonist and cPLA_2_ inhibition decreased parasite burden significantly (*P* < 0.001) (Figure 4(b)). These results together demonstrate that PPAR activation by these agonists and cPLA_2_ inhibition did not increase parasite load.

### 3.5. PPAR Activation by Agonists Selectively Regulates Prostaglandin Production in *L. mexicana*-Infected Macrophages

**Figure 5:**
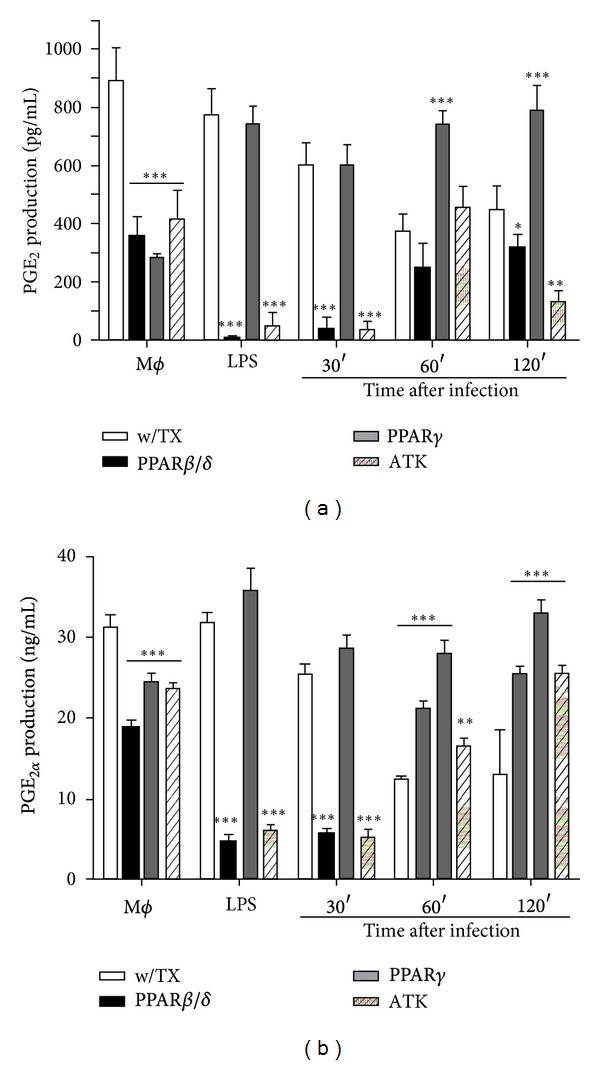
Prostaglandin production by *L. mexicana*-infected macrophages. Prostaglandins were analyzed by MS/MS assay; product scanning experiments were conducted using nitrogen as collision gas, and the collision energy was optimized for individual compounds to generate the most abundant product ions. These product ion spectra were then used to select the precursor-product ion pairs for the development of MRM assays. Deuterium-labeled prostaglandins were used as internal standards for quantitation. Graph bars are mean ± SEM of three independent experiments, and statistical analysis was done comparing, for each time, treated versus nontreated macrophages; (*) *P* < 0.05, (**) *P* < 0.01, and (***) *P* < 0.001.

Prostaglandins are potent ligands of the intracellular PPAR receptors in macrophages, and their binding to PPAR*α* and PPAR*γ* causes macrophage deactivation. Pérez-Santos and Talamás-Rohana [14] have demonstrated that COX-2 inhibition induced leishmanicidal activity by splenocytes [14]. Thus, one possible mechanism of intracellular survival of *Leishmania* is the deactivation of macrophages by prostaglandins produced [27]. In order to investigate if PPAR agonists could modulate inflammatory prostaglandins, macrophages were treated with PPAR agonists or cPLA_2_ antagonist before the infection to look for PG's metabolites in the conditioned media (Figure 5). PPAR activation and cPLA_2_ inhibition decreased significantly 6k-PGF_1*α*_, PGE_1_, and PGF_1*α*_ production (Supplementary Figure 4); however, PPAR*γ* agonist was not able to reduce PGE_2_ production which increased significantly after infection (*P* < 0.001) at 60 and 120 min, respectively (Figure 5). PPAR*β*/*δ* agonist and cPLA_2_ antagonist did not increase PGE_2_ production; even its production diminished (*P* < 0.05 and *P* < 0.001) at 120 min after infection, respectively; moreover, PPAR agonists and cPLA_2_ antagonist significantly increased PGF_2*α*_ production (*P* < 0.001) at 60 and 120 min after infection, respectively. In summary, PPAR agonists and cPLA_2_ antagonist diminished 6k-PGF_1*α*_, PGF_1*α*_, and PGE_1_, and neither *Leishmania* infection nor LPS was able to recover their production; however, PGF_2*α*_ production was increased after infection, and only PPAR*γ* activation increased PGE_2_ production.

### 3.6. PPAR Activation and cPLA_2_ Inhibition Increase the Oxidative Burst during J774A.1 Macrophages Infection with *L. mexicana* Promastigotes

**Figure 6:**
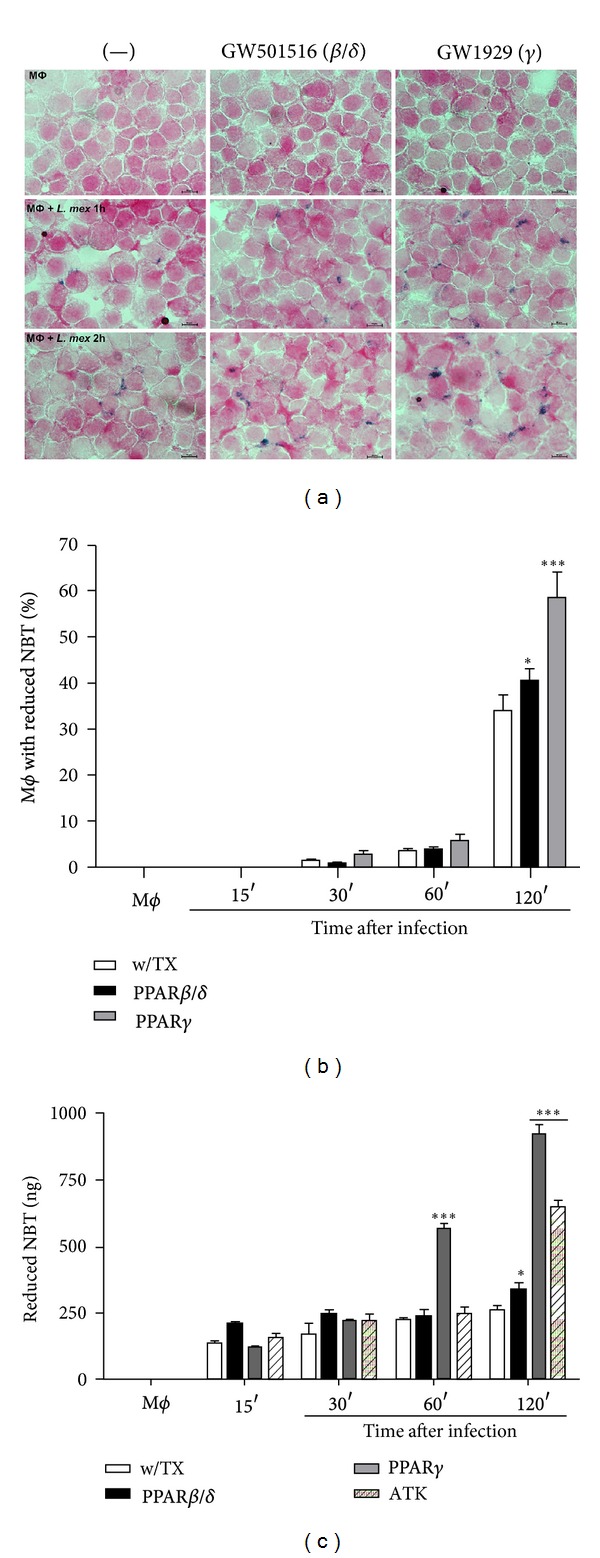
Oxidative burst of *L. mexicana*-infected macrophages. Cells were treated with PPAR agonists 24 h before infection, and the oxidative burst was determined by NBT reduction. NBT was added simultaneously with promastigotes. (a) After the indicated times after infection, slides with infected macrophages were washed and stained for 30 min with Fuccina. Microphotographs show positive cells to NBT reduction in comparison with control cells, which were treated or not with agonists in the presence of NBT. (b) The graph shows percentage of cells positive to NBT reduction. (c) Quantitative analysis of NBT reduction of macrophages infected and treated or not with PPAR agonists. Graph bars are mean ± SEM of three independent experiments, and statistical analysis was done comparing, for each time, treated versus nontreated macrophages; (*) *P* < 0.05, (**) *P* < 0.01, and (***) *P* < 0.001.

Several studies have demonstrated that ROS modulate arachidonic acid metabolism and production of eicosanoids in activated macrophages [28, 29]. In murine and human macrophages, it has been established that the respiratory burst of the cell with production of ROS, such as H_2_O_2_ and O_2_^−^, is primarily responsible for parasite control, as these molecules have been reported to be fatal for *Leishmania* promastigotes. We next analyzed the effect of PPAR agonists and cPLA_2_ antagonist on the oxidative burst induced by *L. mexicana* on J774A.1 macrophages (Figure 6). We found that positive macrophages to *L. mexicana*-induced oxidative burst increased from ~34.1 to 58.6% (*P* < 0.001) by PPAR*γ* agonist at 120 min after infection compared to non-treated macrophages, whereas treatment with PPAR*β*/*δ* agonist increased from ~34.1 to 40.32% (*P* < 0.05) (Figures 6(a) and 6(b)). PPAR*β*/*δ* agonist slightly increased the number of positive oxidative burst macrophages at 120 min after infection, and when the oxidative burst was quantified, it increased, ~1.3-fold at 120 min after infection too (*P* < 0.05) (Figure 6(c)). On the other hand, cPLA_2_ inhibition increased ~2.51-fold the oxidative burst at 120 min after infection (*P* < 0.001) compared to nontreated macrophages (Figure 6(c)). Finally, PPAR*γ* agonist increased the oxidative burst ~2.52-(*P* < 0.001) and ~3.55-fold (*P* < 0.001) at 60 and 120 min after infection, respectively; indicating that PPAR*γ* activation induces an aggressive oxidative response to intracellular parasites (Figure 6(c)).

## 4. Discussion

The cPLA_2_ activation, COX-2 expression, and PG production are positioned at the core of a common regulatory circuit controlling the initiation, magnitude, duration, and resolution of the inflammatory response. During the inflammatory phase, proinflammatory genes expression is controlled at transcriptional, posttranscriptional, and translational levels. According to several reports, in this work we have confirmed that phosphorylated cPLA_2_ and COX-2 are key enzymes during *Leishmania* infection [14, 23, 30]; in addition, we have shown that PPAR activation by agonists prevents cPLA_2_ phosphorylation and COX-2, either protein or mRNA expression, during macrophages infection with* L. mexicana*. Previously, Pérez-Santos and Talamás-Rohana [14] showed that COX-2 inhibition increased IL-12 and IFN*γ* production and induced NO production and parasite killing. Moreover, it has been demonstrated previously that p-cPLA_2_ activates COX-2 and proinflammatory cytokine genes expression through PPAR*γ* response elements [31]; thus, inhibition of cPLA_2_ phosphorylation suppresses those genes [32]. In this context, inhibition of cPLA_2_ phosphorylation with ATK antagonist significantly reduced the mRNA expression of COX-2.

We have demonstrated in this work that PPAR activation by agonists and cPLA_2_ inhibition by antagonist ATK are able to downregulate IL-10 expression throughout the course of infection with *L. mexicana*. It has been demonstrated that cells from IL-10^−/−^ mice produced more NO, IFN*γ*, and IL-12 compared with cells from BALB/c mice [8] and IL-10^−/−^ mice which become resistant to infection [7] suggesting that IL-10 increases susceptibility to *L. mexicana* or *L. amazonensis* infection by inhibiting effector cell functions required for parasite killing. IL-10 inhibition after treatments has several consequences. On one hand, it has been demonstrated that it induces the cPLA_2_-COX-2 pathway; however, results in this work show its downregulation. On the other hand, IL-10 alone or in concert with other molecules activates distinct transcriptional programs that promote the alignment of adaptive responses in a type I or type II direction, as well as by expressing specialized and polarized effector functions [24]. In this case after treatments, infected macrophages did not induce IL-10 expression and remained as classically activated macrophages; this activation program is characterized by TNF-*α*, IL-1*β*, and IL-6 expression, these cytokines being responsible of the oxidative burst [15].

Although TNF-*α* and IL-1*β* have been shown as detrimental in various pathologies, in this work, they are required to sustain classical macrophage activation combined with a small IL-10 production; several reports have demonstrated that, after *Leishmania* infection, TNF-*α* and IL-1*β* induce the phagocytes' NADPH oxidase, whereas IL-10 production inhibits the oxidative stress [15].

The heterogeneity in macrophage phenotypes has given place to its classification as M1 and M2 phenotypes corresponding to classically and alternatively activated macrophages, respectively [33, 34]. M1 macrophages produce high levels of proinflammatory cytokines TNF-*α*, IL-1, IL-6, IL-23, and ROS; M2 macrophages upregulate scavenger, mannose, and galactose receptors and IL-1 receptor antagonist and downregulate IL-1*β* and other proinflammatory cytokines [35]. Available information suggests that classically activated M1 macrophages are potent effector cells integrated in Th1 responses, which kill microorganisms and tumor cells and produce copious amounts of proinflammatory cytokines. In this work, we demonstrated that PPAR activation modulates, selectively, different molecules suggesting a macrophage polarization from M2 to M1 profile; among these molecules, we emphasize IL-10 down regulation and upregulation of IL-6, IL-1*β*, and TNF-*α*. PPAR activation also diminished cPLA_2_ phosphorylation and COX-2 expression. This is in agreement with reports showing that the differentiation into classically activated M1 macrophage increases in cPLA_2_ knockdown cells, whereas the differentiation into alternatively activated M2 macrophage was suppressed by cPLA_2_-knockdown [36]. These findings suggest that cPLA_2_ is involved in regulation of macrophage differentiation and macrophage polarization. Polarized macrophages differ in terms of receptor expression. M2 macrophages are characterized by MR (CD206) expression [37], whereas TLR4 expression is associated with M1 macrophages [24, 38]. Our results show that PPAR activation and cPLA_2_ inhibition significantly increased the TLR4 expression after infection compared to nontreated macrophages, indicating macrophage polarization to M1 profile.

Recent evidence suggests that PPAR**γ** activation may increase the replication of parasites as well as maintain the survival of the host. In particular, PPAR activation has been associated with parasite survival and increase of parasite burden [25, 26, 39, 40]. Flow cytometry analysis revealed that phagocytic activity was not affected by treatments as indicated by zymosan particles assay, and neither PPAR activation nor cPLA_2_ inhibition increased significantly the percentage of infected macrophages or the parasite burden as other agonists do.

Previous studies have reported that *Leishmania* infection, both *in vitro* and *in vivo*, conducts to PGE_2_ production, and it has been postulated that this may favor *Leishmania* persistence and progression [14, 30]. Among prostaglandins analyzed, 6k-PGF_1*α*_, PGE_1_, and PGF_1*α*_ production was increased after infection, and PPAR agonists and cPLA_2_ antagonist diminished their production after infection; however, both PGE_2_ and PGF_2*α*_ production was diminished after infection and increased in treated and infected macrophages. This result may seem in conflict with previous data reporting an increase in PGE_2_ after infection. This could be explained by the fact that COX-2 enzyme and its principal catalytic product PGE_2_ are often equated with inflammation and pathology, a notion fueled primarily by a strong induction of COX-2 expression at sites of inflammation and tissue injury [41]; however, at a later phase, COX-2 promoted resolution by generating an alternate set of reportedly anti-inflammatory prostaglandins through a process now regarded as “eicosanoid class switching.” In addition, it has been demonstrated that PGE_2_ can modulate various steps of inflammation; at the beginning it can induce the expression of COX-2; however, as the inflammation progresses and recovering initiates, PGE_2_ can also inhibit the expression of this enzyme. Therefore, PGE_2_ can exert both proinflammatory and anti-inflammatory effects. Akarasereenont et al. [42] demonstrated that, in HUVEC cells treated with IL-1*β*, PGE_2_ can inhibit COX-2 but not COX-1 protein expression. Therefore, results, suggested that PGE_2_ can initiate a negative feedback regulation in the induction of COX-2 elicited by IL-1*β* in endothelial cells [42]. Based on these results we propose that the expression of IL-1*β* and TNF-*α* maintains COX-2 expression in untreated macrophages and as stated by Akarasereenont et al., PGE_2_ is able to inhibit COX-2 expression in the presence of these proinflammatory cytokines; together with these results, cPLA_2_ inhibition also inhibits COX-2 expression via PPAR*γ* [29], and authors have proposed that cPLA_2_ inhibition can be reverted during M2 to M1 polarization of macrophages [36]. All these results may explain why, during PPAR*γ* activation, PGE_2_ production increased during macrophages infection with *L. mexicana*.

In murine and human macrophages, it has been established that the respiratory burst of the cell, with the production of ROS such as H_2_O_2_ and O_2_, is largely responsible for parasite control as these molecules have been reported to be fatal for *Leishmania* promastigotes [43, 44]. It has been demonstrated that* L. donovani* inhibits the respiratory burst in macrophages [45]. In this work, we have shown that PPAR activation, as well as cPLA_2_ inhibition, increased ROS production by 1-2 folds. It has been demonstrated that long-chain fatty acids increase intercellular ROS synthesis via PPAR*α*, and its inhibitors reduced ROS concentration [46].

The FDA has approved several synthetic PPAR ligands as therapeutic drugs [47]. These PPAR ligands could have a potential use in parasitic diseases. Recently, Serghides et al. [48] have shown that rosiglitazone, a PPAR**γ** agonist, is useful in alleviating cerebral malaria in a murine model [48].

It has been demonstrated that different *Leishmania* species can induce a different profile of cytokines [6, 49], and the enzymes responsible for ROS production are regulated by those cytokines; therefore, treatment against *Leishmania* infection would depend on the infecting species. Thus, cutaneous leishmaniasis caused by *L. major* could be alleviated with PPAR*α* and PPAR*γ* ligands in murine models [26, 39]. In this work, we have demonstrated that PPAR*β*/*δ* and mainly PPAR*γ* activation induced macrophage activation through their polarization to M1 profile, with an increase of microbicidal activity against an intracellular pathogen, *L. mexicana*. Based on the above reasons, macrophage polarization from M2 to M1 through PPAR activation in the presence of agonists could be considered as a potential signaling pathway for drug design and eventually to be used as a strategy to control intracellular parasitosis.

## Supplementary Material

Supplementary Figure 1. COX-2 mRNA expression post-infection; Supplementary Figure 2. Cytokines mRNA expression post-infection; Supplementary Figure 3. MR expression in *L. mexicana* infected macrophages; Supplementary Figure 4. Prostaglandin production by *L. mexicana* infected macrophages.
