# Supplementary material for: PPAR Activation Induces M1 Macrophage Polarization via cPLA2-COX-2 Inhibition, Activating ROS Production against Leishmania mexicana
Source: Biomed Res Int. 2013 Feb 28;2013:215283. doi: 10.1155/2013/215283 (PMC3600276; doi:10.1155/2013/215283)
Supplement: Supplementary file 1 — Supplementary Figure 1. COX-2 mRNA expression post-infection; Supplementary Figure 2. Cytokines mRNA expression post-infection; Supplementary Figure 3. MR expression in L. mexicana infected macrophages; Supplementary Figure 4. Prostaglandin production by L. mexicana infected macrophages. [file 215283.f1.docx]

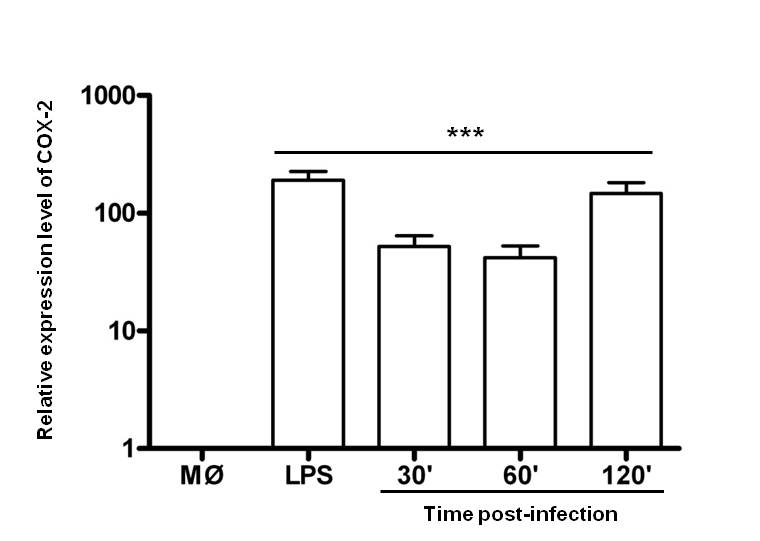


**Supp Figure 1. COX-2 mRNA expression post-infection**

COX-2 mRNA expression in non treated macrophages was evaluated by qRT-PCR and analyzed by 2^-ΔΔC^_T_ method; The *X*-axis intercepts the *Y*-axis at ‘1’ to show the increase of COX-2 expression post-infection. Results are representative of three independent experiments. Graph bars are mean ± SEM of three independent experiments, and Student’s *t*-test analysis was performed comparing to non infected macrophages (***) *P*<0.001.


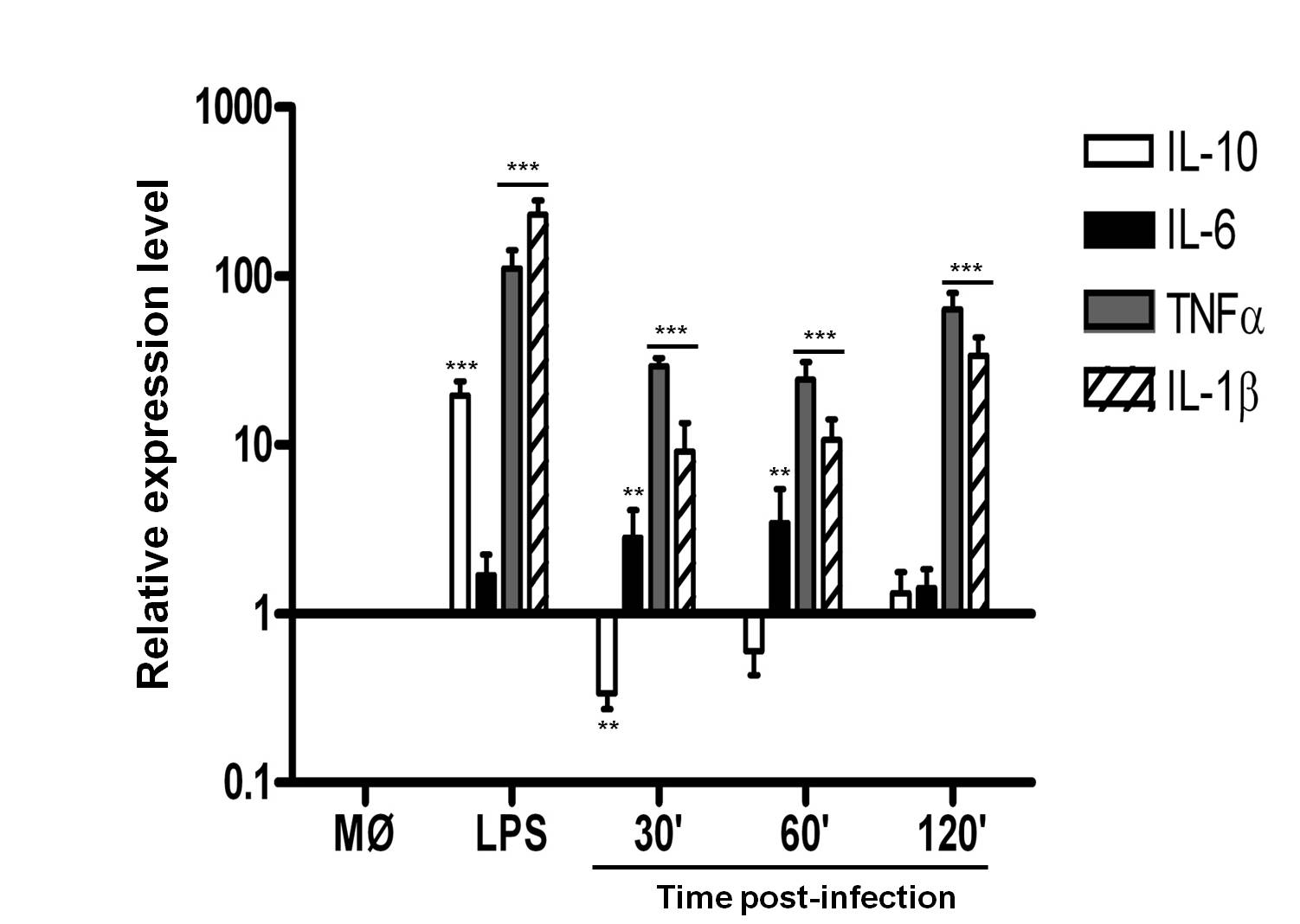


**Supp Figure 2. Cytokine mRNA expression post-infection**

Cytokine mRNA expression was evaluated by qRT-PCR and analyzed by 2^-ΔΔC^_T_ method; The *X*-axis intercepts the *Y*-axis at ‘1’ to show increase or decrease of cytokines post-infection or LPS stimulation comparing vs non-infected or non-stimulated macrophages. Results are representative of three independent experiments. Graph bars are mean ± SEM of three independent experiments, and Student’s *t*-test analysis was performed for each one, comparing vs non infected macrophages; (**) *P*<0.01, (***) *P*<0.001.


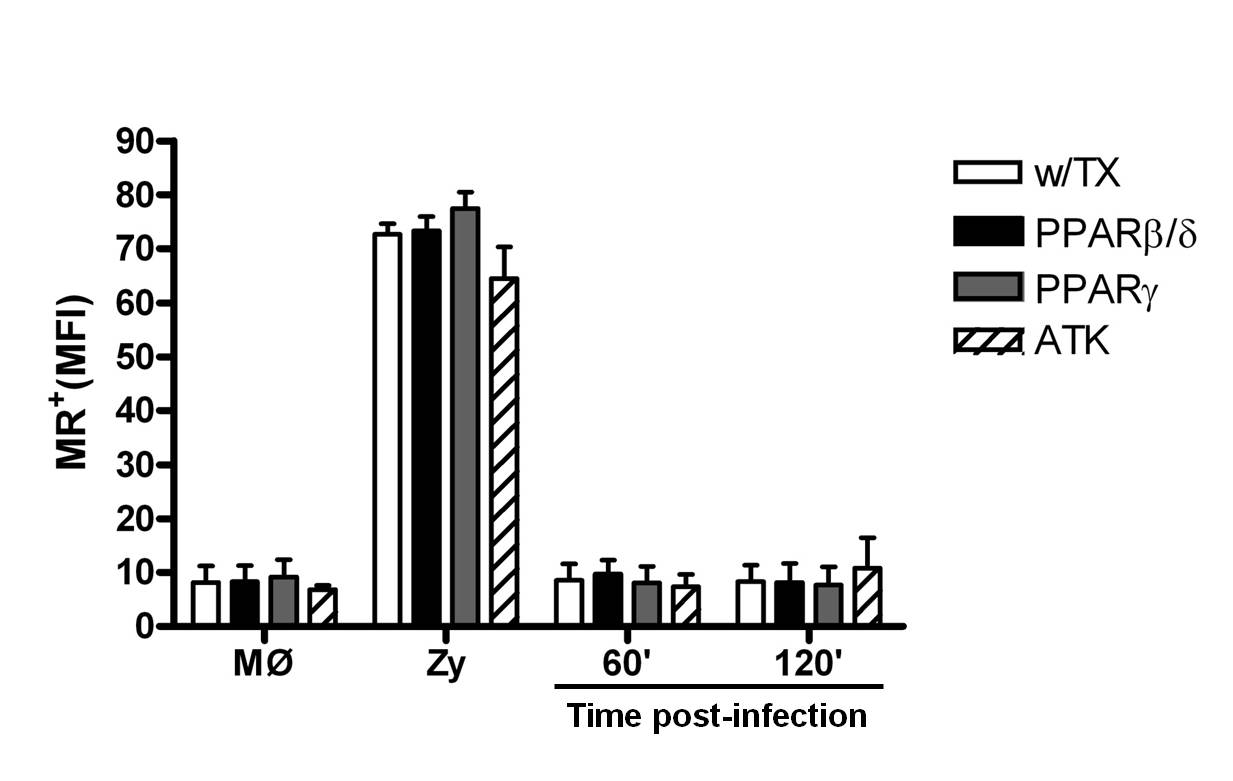


**Supp Figure 3. MR expression in *L. mexicana* infected macrophages.** Cells were treated or not with PPAR’s agonists for 24 h and cPLA_2_ antagonist for 1 h before infection. MR expression was analyzed by flow cytometry; zymosan particles (2 h) were used as a positive control of induction. Graph bars are mean ± SEM of three independent experiments, and statistical analysis was performed comparing, for each time, treated vs non-treated macrophages.


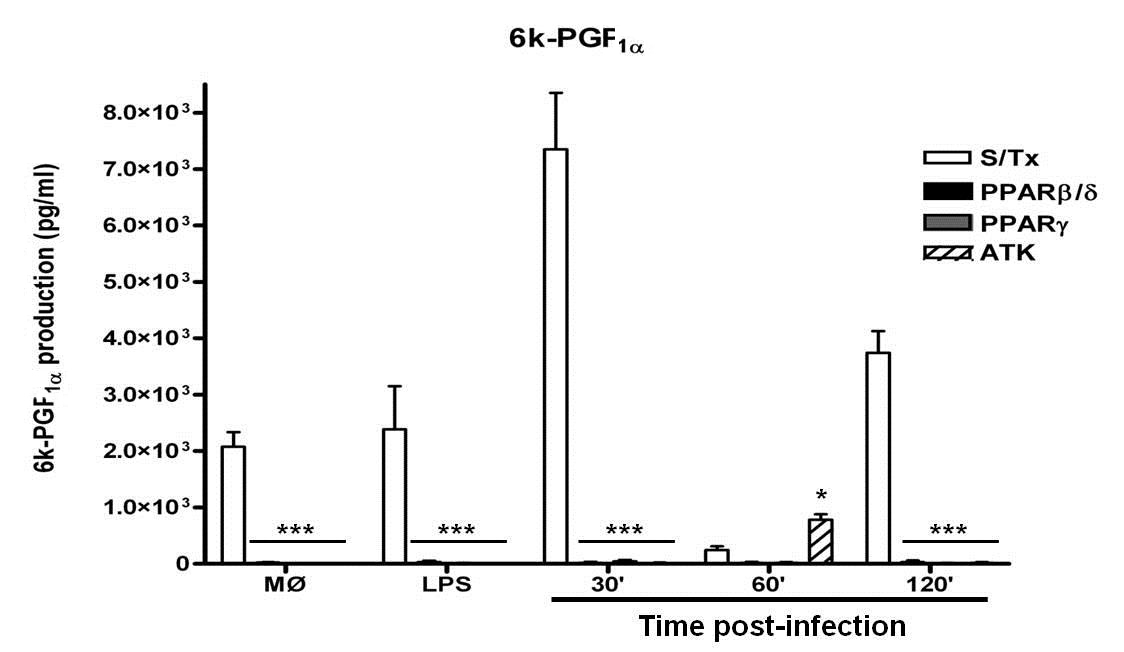


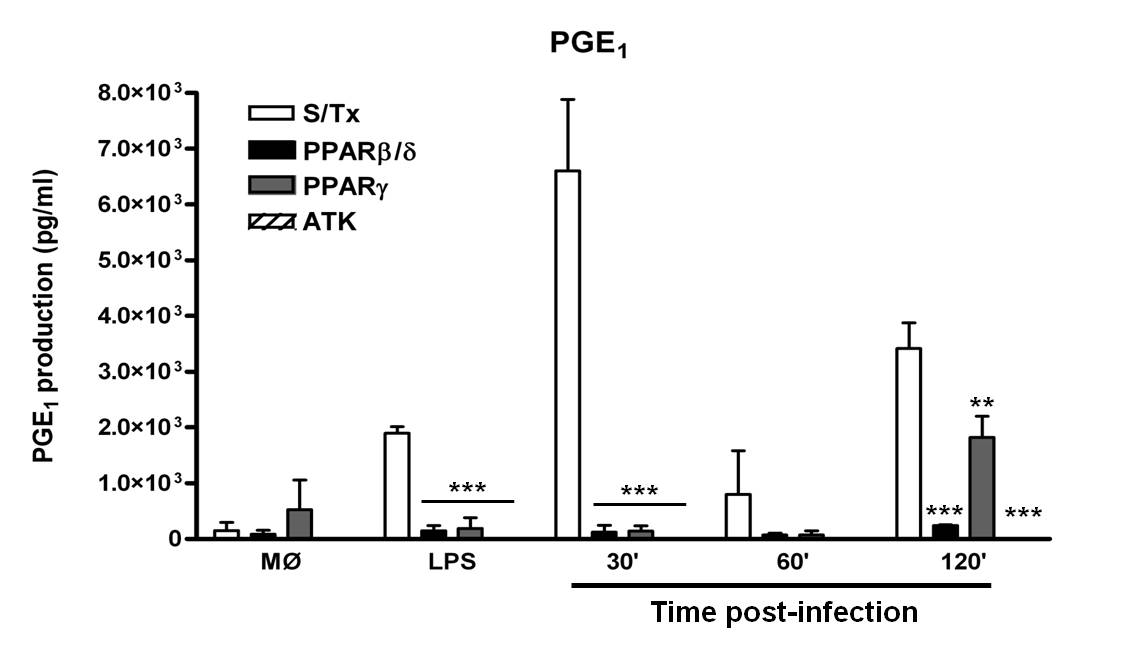


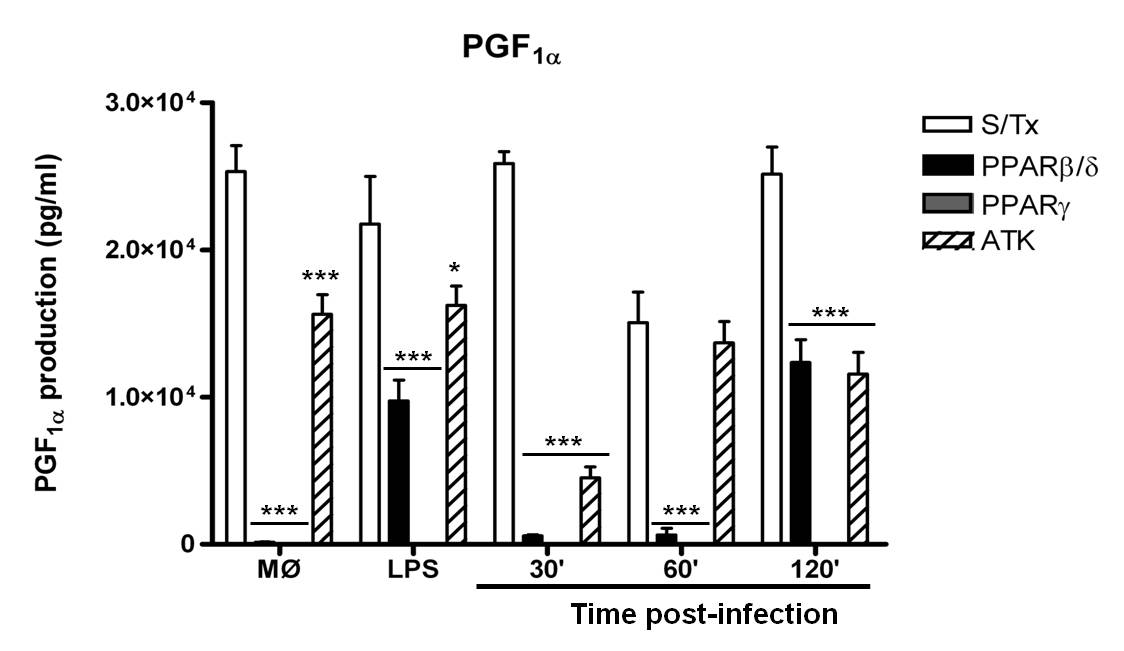


**Supp Figure 4. Prostaglandin production by *L. mexicana* infected macrophages.** Prostaglandins were analyzed by MS/MS assay; product scanning experiments were conducted using nitrogen as collision gas and the collision energy was optimized for individual compounds to generate the most abundant product ions. These product ion spectra were then used to select the precursor-product ion pairs for the development of MRM assays. Deuterium-labeled prostaglandins were used as internal standards for quantitation. Graph bars are mean ± SEM of three independent experiments, and statistical analysis was done comparing, for each time, treated vs non-treated macrophages; (*) *P*<0.05, (**) *P*<0.01, (***) *P*<0.001.
